# Notes and News

**Published:** 1891-01-31

**Authors:** 


### Notes and News.

Collected and Communicated.

At the meeting of the Hospitals Association, held on the 21st inst., Mr. L. Bristowe read a paper on "The Legal Restrictions on Gifts to Charities." A report of this interesting paper and the discussion which followed is unavoidably held over until next week.

As it was considered desirable that the women's balcony in the Louth Hospital should be enclosed with glass, like the men's balcony, the matron, Miss Bradwell, formed a small committee to consider what would be the best means of raising the necessary funds. A drawing-room entertainment and conversazione was decided on, and, thanks to the assistance of friends, their venture proved a great success, and it is hoped that the financial result will form a substantial contribution to the fund now started for the purpose of completing this improvement to the hospital.

The annual entertainment to the patients in the King's College Hospital was given this year as usual. All the patients who were strong enough to sit up, had seats found for them in the great hall, and as many others as could be moved were provided with couches in the galleries. The wards were artistically decorated, and looked very bright and pretty. Instrumental music was played by the Ella orchestra, and among those who gave their services were: Messrs. Hayden Coffin, E. T. Lonnen, George Giddens, and Arthur Playfair. Miss Mary Rorke and Miss Price also contributed to the evening's amusement.

The annual meeting of the subscribers to the "Robertson-Stewart Hospital" (non-infectious cases) was held lately in the Town Hall, Rothsay. Mr. Peter Stewart (Treasurer) submitted the financial statement, which showed that the total income, including a balance of £80 2s. brought forward, amounted to £192 14s. 3d., and the expenditure to £159 2s. 11d., leaving a balance on hand of £33 11s. 3d. The Chairman said that their funds had never been so low as at present. They had only £33 in the bank, with a deduction of eight guineas, which they only held as trustees for the people of Kingarth, and which they could not spend. Under these circumstances they had to consider what was best to be done, as they could not go on for another year with so small a sum.

The following changes have taken place in the medical staff of the Royal Free Hospital consequent upon the retirement, after thirty-seven years' service, of Mr. F. J. Gant, the senior surgeon: Mr. Gant has been appointed consulting surgeon. Mr. James Berry, the senior assistant surgeon, has been appointed full surgeon. Mr. E. W. Roughton has been appointed assistant surgeon, with care of out-patients; and Mr. Willmott H. Evans (M.D.Lond., F.R.C.S.Eng., B.S. and B.Sc.Lond., &c., &c.) has been appointed registrar in succession to Mr. Roughton. Mr. J. W. Ernest Solly, F.R.C.S., M.B.Lond. (hon.), L.R.C.P., and L.S.A., has been appointed senior resident medical officer in succession to Mr. Ewen C. Stabb (resigned).

On the 10th inst. the Hull Royal Infirmary Working Men's Committee held their thirtieth annual meeting, presided over by Mr. Russell. Mr. G. Ramsay, the Secretary, read the report, which showed that the operatives' donations for 1890 amounted to £1,535 3s. 3d., being an increase of £122 0s. 3d. over the year 1889. Mr. O'Brian moved, and Mr. Still seconded, the adoption of the report. Alderman Symons remarked that the exertions of the committee had been very successful during the past year, and he hoped that good relations would continue to exist between the capitalists and the working classes, so that their work as a Committee might be even more successful during the current year. Captain Lempriere said he need not assure them how highly the work done by the Working Men's Committee was appreciated by the Board of Management. He wished them increased success in their labour.

The Chairman of the Finance Committee of the Leicester Infirmary made a statement at the quarterly meeting of Governors, which was exceptionally important for two things. In the first place, it was announced that the expenditure had exceeded the income by £1,600, of which about half had been on the ordinary account. Secondly, as about £1,200 had been spent on the typhoid fever patients, the Chairman strongly recommended that the Board should declare for a totally new departure. This was that no additional borough patients should be admitted except under a written guarantee that the Town Council would defray all expenses, and no county case after January 1st next. The Chairman justified this radical difference by the fact that the borough subscriptions to the Fever House barely reached £10, while the receipts from the county constituted the principal revenue. In the event no resolution was adopted. It is to be hoped that this year the subscriptions to the hospital will increase, instead of declining, as during the past year.

A special Court of the Governors of the Royal Berks Hospital was held on the 30th ult. The Chairman (Major Thoyts) said that this court had been summoned according to the resolution of the Board of Management of December 9th, to consider whether they should adopt the recommendation of the sub-committee to make some slight alteration in regard to the admission of in-patients, for the convenience of those who came to the hospital. It was thought advisable that in-patients should be admitted by ticket on other days besides Tuesdays, and a resolution would be moved accordingly; and to do that properly, and in order, some slight alteration would be required in the rules. Mr. T. H. Wilson then proposed on behalf of the Board of Management: "That in-patients be admitted on Saturdays at twelve o'clock in the same way as on Tuesdays, and that in-patients be discharged on Thursdays, as well as on Tuesdays and Saturdays." The proposal has the entire approval of every member of the medical staff, who met the Committee specially appointed to consider the matter. The Rev. E. A. Gray seconded the resolution, which was agreed to, and the necessary alteration in the rules were accordingly made.

BY permission of Sir Henry and Lady Layard, a drawing-room sale will be held at 1, Queen Anne Street, Cavendish Square, on Tuesday, the 27th inst., in aid of the endowment of the Petersburg Cot at the Hospital for Sick Children in Great Ormond Street.

THE annual report of the Woman's Branch of the Worcester Convalescent Fund, which was read at the meeting in the Guildhall, shows that a great amount of good has been done during the last twelve months. There are now 782 members, 152 of whom have joined during the last year. Twenty-four members received assistance to enable them to visit various sanatoriums and baths. From some news was received of complete recovery, and others were greatly benefited. The total income for the year, including a balance from last year of £38 0s. 9d., was £92 15s. 3d. The expenditure amounted to £52 6s. 9½d., of which £37 4s. 10½d. was for assistance to members, leaving a balance in hand of £40 8s. 5½d.

THE annual meeting in connection with the Worcester Ophthalmic Hospital, was held on the 20th inst. Captain Leggett was in the chair. The Committee reported that the total number of patients during the past year had been 717, of whom 658 were out-patients, and 59 in-patients. The total income from every source, including legacies, was £264 18s. 3d., against £272 17s. 3d. in 1889. A further investment of £100 had been made, and the capital of the hospital now stood at £1,250. The Chairman, in moving the adoption of the report, said that it compared favourably with the reports of previous years. The institution was doing good work quietly, and deserved public support.

THE severe weather has not been without its baneful influence on the patients of the Royal Hospital for Incurables, on Putney Hill. An unusually large number of poor sufferers have died during the last six weeks, and more patients are entirely confined to their beds than on an average. Such, however, as were able to be up, were brought into the large drawing-room on the night of the 20th, where they were made to forget their helplessness and pain for a little while, in the novel amusement of seeing a magic lantern. The slides consisted of 240 very beautiful photographs of many places which the patients could read of but could never see. Their interest never flagged, and the little grateful speech by one of the patients, at the close of the entertainment, was echoed right heartily by the whole assembly.

DR. J. ROBERTS THOMSON, Chairman of the Committee, presided at the annual meeting of Governors of the Royal Victoria Hospital, Bournemouth. The report, which was taken as read, made suitable reference to the formal opening of the hospital by the Prince of Wales on January 16th, and the visit of the Duchess of Albany on Easter Tuesday; and stated that among other results of the Royal visit Sir Edward Guinness had become a Life Governor of the institution, and had forwarded a cheque for £100. During the past year 2,303 patients have been treated, of which number 226 were in-patients. The subscriptions to the maintenance account amounted to £1,050, and the Hospital Sunday collections and collection at the banquet given on the occasion of the Prince of Wales' visit brought up the total receipts to £1,897 5s. 9d. The total expenditure was £1,972 19s. 6d., leaving a balance due to the treasurer of £75 13s. 9d., so the necessity for increased reliable income was apparent. On an average 20 beds were always occupied, which showed the great amount of relief daily administered to the sick poor. Every possible economy is practised in working the hospital. On the motion of the Chairman two alterations in the rules were then made: (1) That one ticket only instead of four be required for the admittance of in-patients, and (2) that the Committee should be enabled to charge "not less than £2 2s. per week" for private patients using the hospital, the rules hitherto having fixed the amount at £2 2s.

